# Thrombospondin-1 Silencing Ameliorates Osteoblastic Differentiation of Aortic Valve Interstitial Cells via Inhibiting Nuclear Factor-*κ*B Pathway

**DOI:** 10.1155/cdr/3845211

**Published:** 2025-04-07

**Authors:** Qing Li, Chengxiang Song, Zisong Wei, Junli Li, Hao Zhou, Shuoding Wang, Hongde Li, Haoran Yang, Qiang Luo, Mao Chen

**Affiliations:** ^1^Laboratory of Cardiac Structure and Function, Institute of Cardiovascular Diseases, West China Hospital, Sichuan University, Chengdu, China; ^2^Cardiac Structure and Function Research Key Laboratory of Sichuan Province, West China Hospital, Sichuan University, Chengdu, China; ^3^National Clinical Research Center for Geriatrics, The Center of Gerontology and Geriatrics, West China Hospital, Sichuan University, Chengdu, China; ^4^Department of Cardiology, West China Hospital, Sichuan University, Chengdu, China

**Keywords:** calcific aortic valve disease, nuclear factor-*κ*B, osteoblastic differentiation, thrombospondin-1, valve interstitial cell

## Abstract

**Objective:** Calcific aortic valve disease (CAVD) is a progressive cardiovascular condition driven by the osteogenic differentiation of valve interstitial cells (VICs), with no effective drug therapies currently available. Hence, our objective is to investigate the impact of thrombospondin-1 (TSP-1) silencing on CAVD progression.

**Methods:** In vitro experiments were employed using human primary VICs with TSP-1 knockdown, cultured in osteogenic induction medium, and followed by analyses including western blot, alkaline phosphatase staining, alizarin red staining, immunofluorescence, and flow cytometry. In vivo experiments used two murine models of CAVD to determine the role of TSP-1 silencing on aortic valve calcification.

**Results:** We observed that silencing of TSP-1 reduced the osteogenic differentiation of VICs. Subsequent experiments demonstrated that TSP-1 knockdown suppressed nuclear factor-*κ*B (NF-*κ*B)–mediated inflammation during osteoblastic differentiation of VICs. Consistent findings were also observed in two murine models of CAVD.

**Conclusions:** The present study has shown that TSP-1 silencing could mitigate the development of CAVD by inhibiting NF-*κ*B-mediated inflammation. We propose that targeting TSP-1-mediated NF-*κ*B pathway could provide a potential therapeutic method for treating CAVD.

## 1. Introduction

Calcific aortic valve disease (CAVD) is the most frequent cardiac valve disorder, featured by the gradual thickening and calcification of the heart valve leaflets, leading to hemodynamic disturbance and subsequent aortic stenosis [1]. Valve interstitial cells (VICs), the most prevalent cell type across all layers of heart valves, are specialized fibroblasts that play a key role in maintaining aortic valvular function [2]. Under certain conditions, VICs transform into osteoblast-like cells, thereby promoting the calcification process associated with CAVD [3]. Currently, there are no available pharmacological treatments to reduce or stop the development of CAVD other than surgical or transcatheter heart valve replacement. Thus, a comprehensive understanding of mechanisms that drive the phenotype of bone-forming osteoblasts of VICs is vital for exploring new therapies for this disease.

Thrombospondin-1 (TSP-1) is a homotrimer glycoprotein from the matricellular protein family, involved in diverse biological processes such as angiogenesis, cell adhesion, proliferation, and atherosclerosis [4, 5]. Previous research has shown that TSP-1 is localized in osteoid and mineralized bone matrix, suggesting its significant role in osteoblastic function [6, 7]. However, the specific role of TSP-1 in VIC-mediated calcification in CAVD remains unexplored. Additionally, the nuclear factor-*κ*B (NF-*κ*B) signaling pathway facilitates the transdifferentiate of VICs into osteoblast-like cells and the formation of calcific nodules [8]. Given that TSP-1 also exhibits anti-inflammatory functions across various diseases processes [9–11], we hypothesize that TSP-1 silencing may attenuate the osteoblastic differentiation of VICs via inhibiting the NF-*κ*B-mediated inflammation pathway, thereby offering a potential therapeutic target for mitigating valvular calcification.

In our present study, we explored the function of TSP-1 silencing in the progression of valvular calcification. We found that TSP-1 silencing inhibited osteogenic differentiation of VICs and alleviated the advancement of CAVD. Mechanistically, TSP-1 silencing attenuated VIC calcification via inhibiting the NF-*κ*B-mediated inflammation pathway. Our results indicate that TSP-1 may be a promising medical target for treating aortic valve calcification.

## 2. Materials and Methods

### 2.1. Human Aortic Valve Samples

Calcific aortic valve leaflets were acquired from patients diagnosed with CAVD, while noncalcific leaflets were acquired from individuals undergoing heart valve replacement due to aortic dissection, dilatation of aortic sinus, and valve prolapse. Noncalcific valve samples were observed by gross and microscopic evaluation of hematoxylin and eosin (H&E)–stained cryosections to identify the missing calcific nodules. The baseline characteristics of enrolled participants are shown in Table S1. The study protocol was conducted with the approval of the Ethics Committee of the West China Hospital of Sichuan University and adhered to the Declaration of Helsinki. Written informed consent was acquired from all individuals prior to participation.

### 2.2. Animal Experiments

Two murine models of CAVD were constructed by direct wire injury (DWI) and high-fat diet induction in our present study. The DWI mouse model was carried out in both male and female 12-week-old TSP-1-deficient mice (TSP-1^−/−^) (*n* = 8) and wild-type C57BL/6J mice (TSP-1^+/+^) (*n* = 8), obtained from Cyagen Biosciences (Suzhou, China). In brief, the spring wire was penetrated into the right carotid artery and then carefully guided into the left ventricle [12]. Valve injury was caused by scratching the aortic leaflets with the wire for 20 times and spinning the wire positioned on the left ventricular side of the valve for 50 times [12]. Sham operation was carried out similarly, but with no wire penetration into the left ventricle. All mice were maintained on a normal diet (ND) for 12 weeks. High-fat diet–induced CAVD model was performed in 4-week-old male ApoE^−/−^ mice sourced from Charles River Laboratory Animal Technology Co. Ltd. (Beijing, China). Briefly, before a high cholesterol diet (HCD) treatment, the adeno-associated virus subtype 9 containing shRNA targeting TSP-1 (AAV9-sh-TSP-1) and AAV9-scramble (negative-control vector, AAV9-scr) (8.0 × 10^11^ vector genomes/per mouse) was injected into ApoE^−/−^ mice through tail vein as previous research described [13]. ApoE^−/−^ mice were then randomly separated into four groups: (1) ND with AAV9-scr (*n* = 5), (2) HCD with AAV9-scr (*n* = 8), (3) ND with AAV9-sh-TSP-1 (*n* = 5), and (4) HCD with AAV9-sh-TSP-1 (*n* = 8). ApoE^−/−^ mice were followed for 24 weeks to observe valvular calcification [13, 14]. Besides, as cluster of differentiation 47 (CD47) is a high-affinity TSP-1 receptor [15], ApoE^−/−^ mice fed an HCD treatment were injected with either 200 *μ*g of the inhibitory anti-CD47 antibodies (BioXcell, United States, *n* = 5) or immunoglobulin G (IgG) control (BioXcell, United States, *n* = 5) via tail vein daily, at the dose previously studied [16]. All animals involved in our study were housed under pathogen-free, temperature-controlled conditions with unrestricted access to food and water, following a 12-h light/dark cycle. Finally, after echocardiographic parameters were assessed by transthoracic echocardiography using a 15∼70 MHZ array probe (MX500D) linked to a Vevo 3100 Imaging system (Fujifilm Visualsonics, Canada), all animals were euthanized. Their heart valves and blood samples were acquired for subsequent examinations. Experiments involving animals were approved by the Animal Care and Use Committee of West China Hospital of Sichuan University, and all procedures adhered to European Communities Council Directives 86/609/EEC and 2010/63/EU.

### 2.3. Human VIC Culture and Treatment

Human primary VICs were obtained from noncalcific aortic valve leaflets as previously described [13, 17]. In brief, the valve leaflets were maintained in 2 mg/mL collagenase II (Thermo Fisher Scientific, United States) at 37°C for 10 min, followed by vortexing to remove aortic endothelial cells. Subsequently, the samples were maintained in collagenase at the same concentration for an additional 4 h. Finally, isolated VICs were cultured in Dulbecco's modified Eagle's medium (DMEM), which was supplemented with 10% fetal bovine serum and 1% penicillin/streptomycin. Cells between passages 2 and 5 were chosen for further experiments. The identification of VICs is presented in Figure S1. To induce calcification, cells were cultured in osteogenic induction medium, which was consisted of DMEM, 5% fetal bovine serum, 1% penicillin/streptomycin, *β*-glycerophosphate (2 g/L, G9422, Sigma-Aldrich, United States), dexamethasone (40 g/L, HY-14648, MCE, United States), and ascorbic acid (50 *μ*g/mL, A4544, Sigma-Aldrich, United States) as previously described [17]. The medium was replaced every 3–4 days and VICs were collected at designated time points, either Day 3, 5, or 21. To knock down TSP-1, VICs were cultured with a compound of TSP-1 siRNA (50 nM) (sense: 5⁣′-CUGCGUUGGUGAUGUAACATT-3⁣′; antisense: 5⁣′-UGUUACAUCACCAACGCAGTT-3⁣′), Lipofectamine RNAiMAX (Invitrogen, United States), and Opti-MEM (Thermo Fisher Scientific, United States) for 8 h. Control VICs were incubated with scrambled siRNA. The siRNA sequences were obtained from GenePharma Co. Ltd. (Shanghai, China). For the following experiments, VICs were treated with active recombinant human TSP-1 (rhTSP-1) (100 ng/mL, 3074-TH, R&D Systems, Minnesota) or NF-*κ*B inhibitor pyrrolidinedithiocarbamate ammonium (PDTC) (164 *μ*g/L, HY-18738, MCE, United States) as previously described [18].

### 2.4. Western Blot Analysis

Protein extracted from VICs was conducted using radioimmunoprecipitation assay lysis buffer with added protease and phosphatase inhibitors. The extracted protein was separated using 10% or 12.5% sodium dodecyl sulfate-polyacrylamide gels and then transferred onto 0.22-*μ*m polyvinylidene difluoride (PVDF) membranes. PVDF membranes were treated with primary antibodies with gentle agitation overnight, followed by anti-rabbit (77 ng/mL, 7074, CST) or anti-mouse (184 ng/mL, 7076, CST) horseradish peroxidase-linked secondary antibodies. The protein–antibody complex was subsequently visualized using enhanced chemiluminescence detection. Primary antibodies against osteocalcin (OCN) (1 *μ*g/mL, ab133612, Abcam), osterix (0.5 *μ*g/mL, ab209484, Abcam), osteopontin (OPN) (0.5 *μ*g/mL, ab214050, Abcam), TSP-1 (0.5 *μ*g/mL, ab267388, Abcam), NF-*κ*B p65 (208 *μ*g/mL, 8242, CST), phosphorylated NF-*κ*B p65 (57 ng/mL, 3033, CST), and GAPDH (1 *μ*g/mL, A19056, ABclonal, Wuhan, China) were used. Finally, band intensities were quantified using Fiji ImageJ software, with the relative density calculated as the ratio of the target gene to GAPDH.

### 2.5. Alkaline Phosphatase (ALP) Staining

The VICs were seeded in 12-well plates. Once they reached 60%–70% confluence, the medium was replaced with osteogenic induction medium. After 21 days of induction, VICs were fixed in 4% paraformaldehyde (PFA) solution. Subsequently, VICs were maintained with ALP (Beyotime, Shanghai, China) solution overnight according to the manufacturer's instructions. Finally, the samples were observed and photographed.

### 2.6. Alizarin Red Staining

For alizarin red staining, cells were seeded in 24-well plates. After 28 days of induction, VICs were washed with phosphate-buffered saline and fixed with 4% PFA solution. Afterwards, cells were exposed to alizarin red S solution (Cyagen Biosciences, Guangzhou, China) for 60 min at room temperature. Finally, excess stain was removed by washing the cells with deionized water.

### 2.7. Immunofluorescence Staining

For immunofluorescence staining, VICs were fixed in 4% PFA solution for 10 min and permeabilized with 0.1% Triton-X for 5 min. Cells were then maintained with 5% bovine serum albumin for 60 min. Following this, the primary antibodies were employed and treated overnight. The primary antibodies included CD31 (5 *μ*g/mL, ab9498, Abcam), Vimentin (0.4 *μ*g/mL, ab92547, Abcam), TSP-1 (2.5 *μ*g/mL), OPN (2.5 *μ*g/mL, 14-9096-82, Invitrogen), and NF-*κ*B p65 (88 ng/mL, 6956, CST). The next day, Alexa Fluor 488–labeled secondary antibody (4 *μ*g/mL, A11034, Thermo Fisher Scientific) or Alexa Fluor 555–labeled secondary antibody (4 *μ*g/mL, A32727, Thermo Fisher Scientific) was treated for 60 min at 37°C. VICs were mounted using antifade reagents with 4⁣′,6-diamidino-2-phenylindole. Imaging was acquired using the Lecia stellaris confocal laser scanning microscope (Germany).

### 2.8. Enzyme-Linked Immunosorbent Assay of Cytokines

Proinflammatory cytokine levels, including interleukin-1*β* (IL-1*β*), IL-6, tumor necrosis factor-*α* (TNF-*α*), and monocyte chemoattractant protein-1 (MCP-1), were quantified using enzyme-linked immunosorbent assay in either human VIC culture supernatants or the serum samples of mice (ABclonal, Wuhan, China). The procedures followed the manufacturer's instructions.

### 2.9. Flow Cytometry

Briefly, VICs were trypsinized from each treatment well. The procedure involved fixation with 4% PFA and permeabilized using 0.5% Triton-X. Then, VICs were maintained with primary antibodies TSP-1 (5 *μ*g/mL) or OPN (20 *μ*g/mL, CL488-22952, Proteintech, Wuhan, China) for 30 min. Afterwards, VICs were stained with Alexa Fluor 488–labeled secondary antibody in the dark. Finally, the mean immunofluorescence intensity was quantified by BD LSRFortessa flow cytometry (United States).

### 2.10. H&E and Von Kossa Staining

Briefly, tissue samples were fixed in 4% PFA and then embedded in paraffin. The samples were sectioned at 5 *μ*m and stained with a standard H&E staining protocol to assess the thickness of aortic valve leaflets, as previously described [13]. Additional sections were stained with 5% silver nitrate solution according to the manufacturer's protocol (Servicebio, Wuhan, China). Von Kossa staining was used to evaluate calcific nodules in aortic valve leaflets as previously described [19].

### 2.11. Immunohistochemistry

About 5-*μ*m paraffin-embedded aortic valve tissues were used for immunohistochemistry staining to detect TSP-1. In brief, after antigen retrieval, the tissues were stained with a primary antibody against TSP-1 (0.5 *μ*g/mL) and then with an anti-rabbit HRP-conjugated secondary antibody (1 *μ*g/mL, ab205718, Abcam). Next, the slides were treated with diaminobenzidine and stained with hematoxylin. The images were obtained using a Leica microscope (Germany).

### 2.12. Statistical Analysis

All data originated from three independent experiments or above. The Shapiro–Wilk test was applied to evaluate the normal distribution of continuous data. Continuous data were displayed as means ± standard error of the mean or median (interquartile range), where applicable. For normally distributed data, Student's *t*-test or two-way analysis of variance followed by Bonferroni's multiple comparison test was used for analysis. For nonnormally distributed data, the Mann–Whitney *U* test or Kruskal–Wallis test was used for analysis. Categorical data were compared using chi-square analysis. Statistical significance was defined as a two-sided *p* value less than 0.05. Statistical analyses were conducted using the Prism version 8.2.1 (GraphPad Software Inc., La Jolla, California, United States) or SPSS software version 25.0 (IBM, Armonk, United States).

## 3. Results

### 3.1. TSP-1 Is Increased in CAVD and Calcific VICs

**Figure 1:**
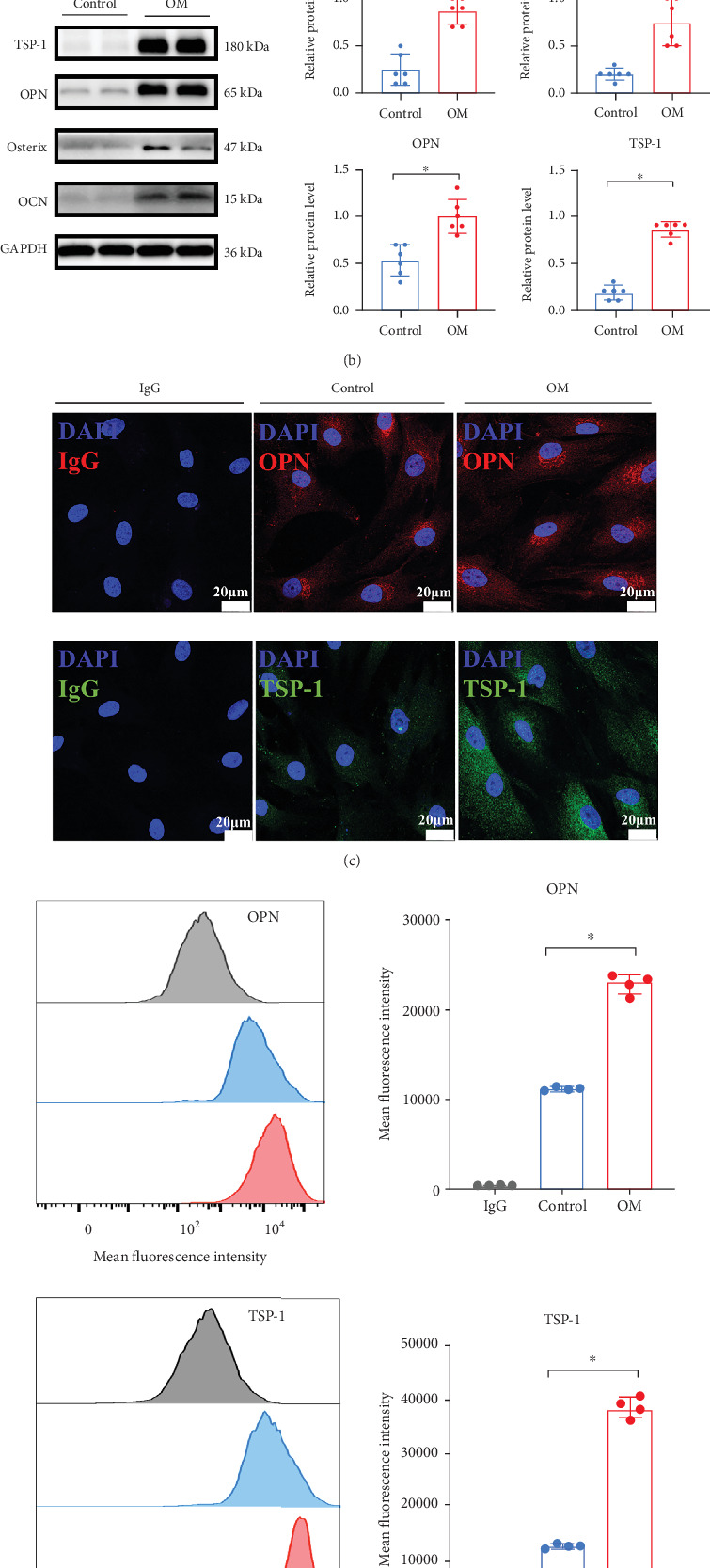
TSP-1 expression is upregulated in CAVD. (a) H&E staining of aortic valve tissues and immunohistochemical staining showed TSP-1 in non-CAVD and CAVD groups (*n* = 4 for each group). (b) Western blot analysis and quantification of OCN, osterix, OPN, and TSP-1 expression in human VICs under calcifying medium (*n* = 6 for each group). (c) Immunofluorescence staining for OPN and TSP-1 in human VICs during the calcification process (*n* = 6 for each group). (d) Flow cytometry for OPN and TSP-1 in human VICs under osteogenic induction medium (*n* = 4 for each group). Data are presented as mean ± SEM and statistical significance was compared by Student's *t*-test. H&E, hematoxylin and eosin; TSP-1, thrombospondin-1; CAVD, calcific aortic valve disease; OCN, osteocalcin; OPN, osteopontin; VICs, valve interstitial cells; OM, osteogenic induction medium; DAPI, 4⁣′,6-diamidino-2-phenylindole; NS, not significant. ⁣^∗^*p* < 0.05.

To initially determine TSP-1 expression in CAVD, we explored TSP-1 expression in noncalcified aortic valve leaflets and calcific aortic valve leaflets. The immunohistochemistry results demonstrated elevated TSP-1 expression in calcific valve leaflets when compared to noncalcified valve leaflets (Figure 1a). VICs cultured under osteogenic induction medium exhibited significantly elevated expression of calcific markers, such as OCN, osterix, and OPN (Figure 1b). Notably, TSP-1 protein expression was also significantly elevated during VIC calcification (Figure 1b). Immunofluorescence staining also showed upregulated TSP-1 expression in calcified VICs (Figure 1c). In line with these results, flow cytometry confirmed the TSP-1 expression increased during VIC calcification (Figure 1d). Collectively, these results indicated elevated TSP-1 in calcified VICs and calcified valve leaflets may play an important role in CAVD.

### 3.2. TSP-1 Silencing Inhibits Osteoblastic Differentiation of VICs

**Figure 2:**
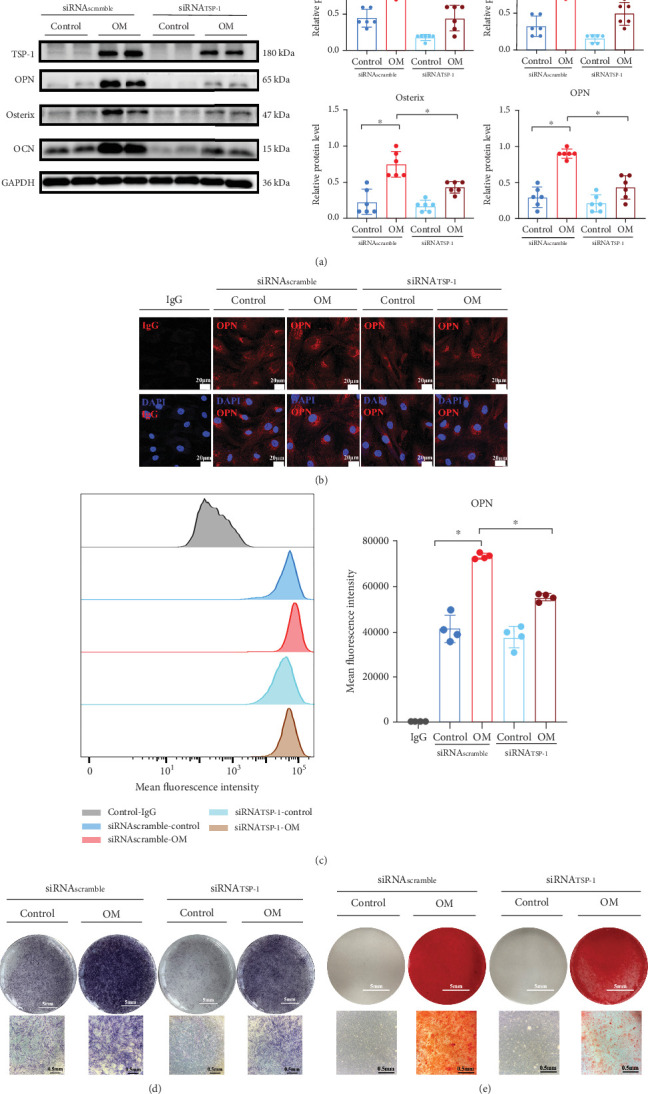
TSP-1 deficiency alleviates osteogenic differentiation of human VICs in vitro. (a) Western blot analysis and quantification of OCN, osterix, OPN, and TSP-1 expression in human VICs following TSP-1 silencing (*n* = 6 for each group). (b) Immunofluorescence staining for OPN in human VICs during the calcification process following TSP-1 silencing (*n* = 6 for each group). (c) Flow cytometry for OPN in human VICs under osteogenic induction medium following TSP-1 deficiency (*n* = 4 for each group). (d) Alkaline phosphatase staining in calcified and noncalcified human VICs following TSP-1 silencing. (e) Alizarin red staining in calcified and noncalcified human VICs following TSP-1 silencing. Data are presented as means ± SEM and statistical significance was compared by two-way analysis of variance followed by Bonferroni's multiple comparison test. TSP-1, thrombospondin-1; VICs, valve interstitial cells; OCN, osteocalcin; OPN, osteopontin; OM, osteogenic induction medium; DAPI, 4⁣′,6-diamidino-2-phenylindole; NS, not significant. ⁣^∗^*p* < 0.05.

To further investigate the function of TSP-1 silencing in CAVD, siRNA-mediated knockdown of the TSP-1 expression was performed in an in vitro VIC calcification model. Western blotting analysis revealed that silencing of TSP-1 in VICs decreased the expression of calcific proteins OCN, osterix, and OPN on Day 5 (Figure 2). Immunofluorescence staining and flow cytometry also demonstrated that TSP-1 silencing reduced OPN expression in VICs cultured under osteogenic induction medium on Day 3 (Figure 2). Additionally, ALP staining revealed a marked reduction in calcific nodule formation in VICs with depleted TSP-1 expression on Day 21 (Figure 2). Similar results were found in alizarin red staining (Figure 2). Collectively, these findings support that TSP-1 silencing is a new inhibitor of osteogenic differentiation of human VICs.

### 3.3. TSP-1 Silencing Inhibits Osteoblastic Differentiation of VICs via the NF-*κ*B p65 Pathway

**Figure 3:**
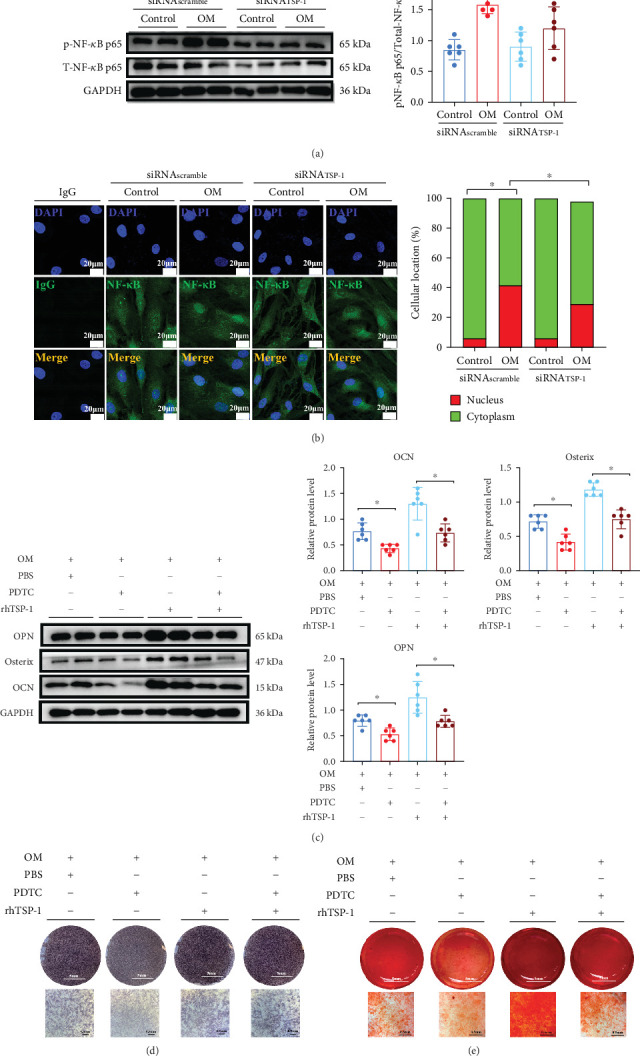
TSP-1 silencing ameliorates osteogenic differentiation of human VICs via inhibiting the NF-*κ*B pathway. (a) Western blot analysis and quantification of pNF-*κ*B p65 and total NF-*κ*B p65 expression following TSP-1 silencing (*n* = 6 for each group). (b) Immunofluorescence staining for the nuclear translocation of NF-*κ*B following TSP-1 silencing (*n* = 6 for each group). (c) Western blot analysis and quantification of OCN, osterix, and OPN expression when human VICs were incubated with rhTSP-1 in the presence of osteogenic induction medium with or without NF-*κ*B inhibitor PDTC (*n* = 6 for each group). (d) Alkaline phosphatase staining of human VICs incubated with rhTSP-1 in the presence of osteogenic induction medium with or without PDTC. (e) Alizarin red staining of human VICs incubated with rhTSP-1 in the presence of osteogenic induction medium with or without PDTC. Data are presented as means ± SEM and statistical significance was compared by two-way analysis of variance followed by Bonferroni's multiple comparison test. pNF-*κ*B, phosphorylated nuclear factor-*κ*B; rhTSP-1, recombinant human thrombospondin-1; VICs, valve interstitial cells; PDTC, pyrrolidinedithiocarbamate ammonium; OM, osteogenic induction medium; DAPI, 4⁣′,6-diamidino-2-phenylindole; NS, not significant. ⁣^∗^*p* < 0.05.

To further investigate the mechanism by which TSP-1 silencing inhibited aortic valve calcification in vitro, we focused on the role of the NF-*κ*B p65 pathway, which has been previously reported to contribute to the transdifferentiation of VICs into osteoblast-like cells [20, 21]. Western blot analysis revealed that TSP-1 silencing in VICs decreased the phosphorylated NF-*κ*B p65 to total NF-*κ*B p65 protein ratio (Figure 3a). Immunofluorescence staining also showed that silencing of TSP-1 expression significantly reduced nuclear translocation of NF-*κ*B p65 in VICs cultured under osteogenic induction medium (Figure 3b). In addition, we found that silencing of TSP-1 expression significantly reduced proinflammatory cytokines, including TNF-*α*, MCP-1, and IL-1*β* levels, but this effect was not detected in IL-6 levels (Figure S2). These findings indicate that TSP-1 silencing can inhibit NF-*κ*B-mediated inflammation. Given our previous findings that NF-*κ*B-mediated inflammation acts as a downstream mechanism of TSP-1 in VIC calcification, we further examined whether inhibiting NF-*κ*B could attenuate TSP-1-induced calcification. To this end, VICs were treated with PDTC, a well-established NF-*κ*B inhibitor, in the presence of osteogenic induction medium and rhTSP-1. Our results demonstrated that rhTSP-1 significantly enhanced NF-*κ*B p65 phosphorylation and promoted its nuclear translocation (Figures S3A and S3B), whereas cotreatment with PDTC markedly attenuated these effects, confirming that PDTC effectively inhibits TSP-1-induced NF-*κ*B activation. Furthermore, western blot analysis displayed that PDTC-mediated NF-*κ*B inhibition blunted the effects of TSP-1 on VIC calcification (Figure 3c), and this was further supported by immunofluorescence staining and flow cytometry (Figures S3C and S3D). Additionally, ALP staining and alizarin red staining confirmed that inhibition of NF-*κ*B with PDTC suppressed TSP-1-induced calcific nodule formation (Figure 3d,e). Together, these results strongly suggest that TSP-1 silencing inhibits VIC calcification via inhibiting the NF-*κ*B signal pathway.

### 3.4. TSP-1 Deficiency Alleviates Aortic Valve Calcification in Mice

**Figure 4:**
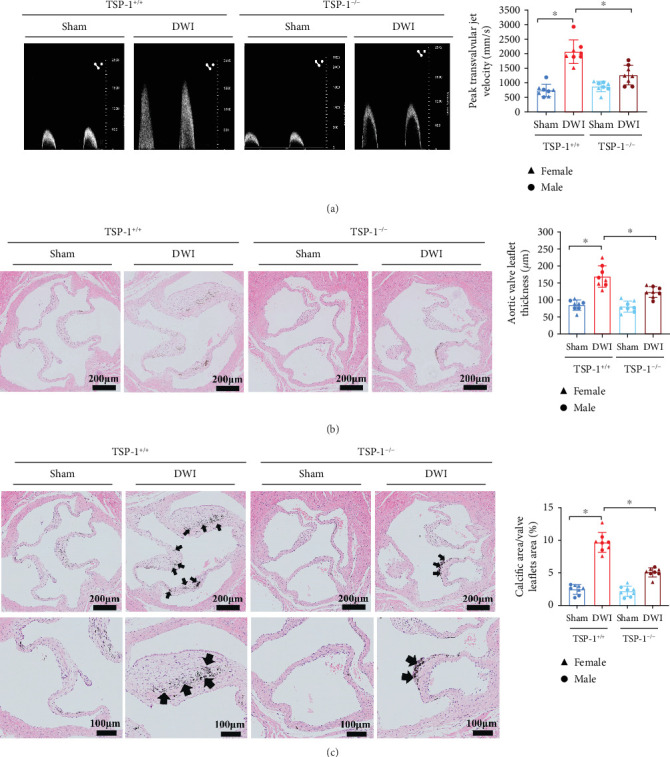
TSP-1 deletion alleviates aortic valve calcification in mice. (a) Echocardiographic data peak transvalvular jet velocity in TSP-1^−/−^ and TSP-1^+/+^ mice (*n* = 8 for each group). (b) H&E staining of aortic valve leaflets from TSP-1^−/−^ and TSP-1^+/+^ mice (*n* = 8 for each group). (c) Von Kossa staining of aortic valve leaflets from TSP-1^−/−^ and TSP-1^+/+^ mice (*n* = 8 for each group). Data are presented as means ± SEM and statistical significance was compared by two-way analysis of variance followed by Bonferroni's multiple comparison test. TSP-1, thrombospondin-1; DWI, direct wire injury; H&E, hematoxylin and eosin; NS, not significant. ⁣^∗^*p* < 0.05.

**Figure 5:**
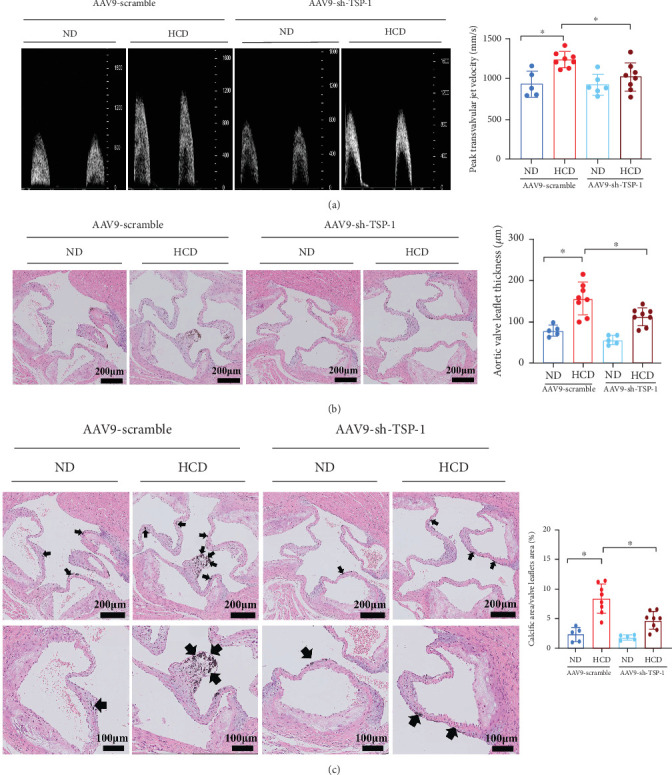
TSP-1 deletion alleviates aortic valve calcification in ApoE^−/−^ mice. (a) Echocardiographic data peak transvalvular jet velocity in ApoE^−/−^ mice (*n* = 5 for ND group, *n* = 8 for HCD group). (b) H&E staining of aortic valve leaflets from ApoE^−/−^ mice (*n* = 5 for ND group, *n* = 8 for HCD group). (c) Von Kossa staining of aortic valve leaflets from ApoE^−/−^ mice (*n* = 5 for ND group, *n* = 8 for HCD group). Data are presented as means ± SEM and statistical significance was compared by two-way analysis of variance followed by Bonferroni's multiple comparison test. TSP-1, thrombospondin-1; AAV9-sh-TSP-1: adeno-associated virus subtype 9 containing shRNA targeting TSP-1; ND, normal diet; HCD, high-cholesterol diet; NS, not significant. ⁣^∗^*p* < 0.05.

To explore the function of TSP-1 in valvular calcification in vivo, two murine models of CAVD were performed using DWI and high-fat diet induction. DWI mouse model was performed in both female and male 12-week-old TSP-1^−/−^ and TSP-1^+/+^ mice. After 12 weeks, the TSP-1^+/+^ mice in the DWI group exhibited a significant increase in peak transvalvular jet velocity (Figure 4a) and aortic valve leaflet thickness (Figure 4b) than those in the sham group. However, TSP-1 knockdown partly restored the change. Moreover, Von Kossa staining displayed increased calcium deposition in the valve leaflets of TSP-1^+/+^ mice following DWI (Figure 4c). Strikingly, TSP-1 silencing partially normalized this change. Besides, consistent results were found in high-fat diet–induced ApoE^−/−^ mice (Figures 5a, 5b, and 5c). We also found TSP-1 silencing in ApoE^−/−^ mice significantly reduces proinflammatory cytokines MCP-1 and IL-1*β* levels, but these effects were not observed in TNF-*α* and IL-6 levels (Figure S4). As previous studies reported CD47 is a high-affinity TSP-1 receptor [15, 22], we used CD47 blockade antibodies and observed that ApoE^−/−^ mice in the anti-CD47 antibody group exhibited reduced aortic valve leaflet thickness, aortic valve calcification, and TSP-1 expression compared to those in the IgG group (Figure S5). These findings may support that TSP-1, acting through CD47, plays a curial role in promoting valvular calcification in ApoE^−/−^ mice.

## 4. Discussion

In this study, we found that TSP-1 expression was upregulated in CAVD. Employing both an in vitro human VIC calcification model and two in vivo CAVD murine models, we provided direct evidence that TSP-1 silencing ameliorated VIC calcification and CAVD progression. Our subsequent experiments demonstrated that TSP-1 depletion suppressed the osteogenic differentiation of VICs through inhibiting NF-*κ*B-mediated inflammation. These results elucidated the function and underlying molecular mechanisms of TSP-1 silencing in the attenuation of valvular calcification.

The major finding of our study is that TSP-1 is a critical molecule involved in osteoblastic activation of VICs, leading to the development of CAVD. TSP-1, a multifunctional homotrimer glycoprotein, belongs to subfamily A of TSP proteins, which includes TSP-1 and TSP-2 [4]. Previous studies reported that TSP-2 is involved in the development of CAVD [23, 24], but data about whether TSP-1 is involved in CAVD is still unknown. TSP-1 participates in multiple biological processes, such as cell–cell interactions, cell proliferation, angiogenesis, and differentiation [4, 5, 25]. In atherosclerotic disease, TSP-1 silencing ameliorates leptin-induced plaque formation in ApoE^−/−^ mice and has been implicated in atherosclerotic plaque progression [10]. Besides, TSP-1 expression is crucial in regulating the development of abdominal aortic aneurysm, with TSP-1-deficient mice showing resistance to abdominal aortic aneurysm formation [26]. In our study, we observed that TSP-1 was upregulated in calcific aortic valves, and silencing of TSP-1 can attenuate CAVD in vitro and in vivo, providing the first evidence of TSP-1's direct involvement in CAVD.

Another key finding of our study is that TSP-1 silencing ameliorates aortic valve calcification via inhibiting the NF-*κ*B inflammation pathway. Using three different mouse models of abdominal aortic aneurysm, Liu et al. found that TSP-1 promotes vascular inflammation, and the inflammation is diminished in aneurysms of TSP-1^−/−^ animals [26]. Similarly, Ganguly et al. found that TSP-1 silencing mitigates leptin-induced inflammatory cell burden in ApoE^−/−^ mice [10]. On the contrary, Moura et al. demonstrated that TSP-1 deficiency leads to high plaque macrophage numbers and accelerates inflammation in the ApoE^−/−^ mice on a normocholesterolemic diet [27]. These controversial findings may reflect the multifunctionality of TSP-1 in different disease processes. In this study, we found that TSP-1 silencing had anti-inflammatory functions in CAVD via inhibiting the NF-*κ*B p65 pathway. Although accumulating evidence indicates that the NF-*κ*B pathway is involved in VIC osteogenic responses [28, 29], the NF-*κ*B pathway in TSP-1-regulated CAVD is unclear. As far as we know, this is the first research displaying that TSP-1 silencing directly inhibits NF-*κ*B-mediated inflammation in human VICs. Our results displayed that TSP-1 silencing suppressed the NF-*κ*B p65 signaling pathway and downregulated NF-*κ*B target genes, including MCP-1, IL-1*β*, and TNF-*α* in VICs. Furthermore, inhibition of NF-*κ*B with PDTC countermanded TSP-1-induced osteogenic differentiation of VICs. These findings exhibit that NF-*κ*B is involved in the crosstalk communication in the TSP-1-induced osteogenic response of VICs.

We further found that ApoE^−/−^ mouse administration with CD47-blocking antibodies had reduced aortic valve thickness and lower expression of TSP-1 (Figure S5). Previous studies have reported that TSP-1 can play differential physiological function via interacting with CD47 [15, 22]. Singla et al. demonstrated that deletion of CD47 attenuates the development of atherosclerosis in hypercholesterolemic mice and further found TSP-1 can mediate activation of CD47 in regulating atherosclerosis development [22]. In Type 2 diabetes mellitus model, high lipid levels induced TSP-1-CD47 activation, and mouse treatment with anti-CD47 antibody had attenuated inflammatory response [30]. Although our study has not further explored the specific mechanism thoroughly, we are tempted to speculate that the TSP-1-CD47 signal is a significant contributor of inflammatory state associated with CAVD.

To our knowledge, no pharmacologic strategies can slow or even halt the progression of CAVD. Cholesterol-lowering drugs were once considered first-line drug candidates for modifying disease progression. Despite promising results in animal experiments [31, 32], several well-designed randomized controlled trials showed that those pharmacologic lipid-lowering drugs failed to blunt the progression of CAVD [33–35]. Therefore, gaining deeper insights into the mechanisms in calcified aortic valvular tissues is important and can provide novel therapeutic regimens for this disease. Our data demonstrated the role of TSP-1 silencing in vitro and in vivo, particularly highlighting its anti-inflammation impactions via inhibiting the NF-*κ*B pathway. TSP-1 might be a novel target for therapeutic intervention for CAVD. Hence, we consider that blocking the TSP-1-mediated NF-*κ*B pathway may help modulate CAVD progression. However, many efforts are needed to implement before findings will be capable to directly help CAVD patients.

### 4.1. Limitations

This research has several limitations. First, cultured human VICs may exhibit altered features compared to those in vivo. However, we validated our findings in vitro using DWI and ApoE^−/−^ mouse models. Second, the more specific mechanisms underlying the role of TSP-1 during valvular calcification were not explored. Further research is required to study the molecular mechanism. Third, we identified TSP-1 silencing ameliorated osteoblastic differentiation of VICs via inhibiting the NF-*κ*B p65 pathway. However, other signaling pathways may be involved in modulating the cellular response to TSP-1. We cannot rule out the possibility that TSP-1 may promote VIC osteogenic responses through additional pathways.

### 4.2. Conclusion

In summary, our study demonstrates that TSP-1 silencing protects against the osteoblastic transformation of VICs by inhibiting NF-*κ*B p65–mediated inflammation. Our findings display that TSP-1 could be a potential therapeutic target for CAVD in humans.

## Data Availability

The data that support the findings of this study are available from the corresponding authors upon reasonable request.

## Supporting Information

Supporting Information Additional supporting information can be found online in the Supporting Information section. Supporting tables and figures were provided to support our results. Table S1. Clinical characteristics of enrolled participants. Figure S1. The identification of human valve interstitial cell. Figure S2. The role of TSP-1 silencing on inflammatory cytokines levels in vitro. Figure S3. TSP-1 silencing alleviates osteogenic differentiation of VICs via inhibiting the NF-*κ*B pathway. Figure S4. The role of TSP-1 silencing on inflammatory cytokine levels in ApoE^−/−^ mice. Figure S5. The role of anti-CD47 on aortic valve calcification in ApoE^−/−^ mice.
