# Supplementary material for: Thrombospondin-1 Silencing Ameliorates Osteoblastic Differentiation of Aortic Valve Interstitial Cells via Inhibiting Nuclear Factor-κB Pathway
Source: Cardiovasc Ther. 2025 Apr 7;2025:3845211. doi: 10.1155/cdr/3845211 (PMC11996288; doi:10.1155/cdr/3845211)

**Supplementary table 1. Clinical characteristics of enrolled participants.**

| Variables | Non-CAVD  (n=4) | CAVD  (n=4) | *P*-value |
| --- | --- | --- | --- |
| Age (years) | 50±4.9 | 52±5.2 | 0.257 |
| Male | 4(100%) | 4(100%) | - |
| Current smoker | 2(50%) | 2(50%) | - |
| BMI (kg/m^2^) | 23.8±2.8 | 24.2±3.0 | 0.423 |
| Hypertension | 2(50%) | 2(50%) | - |
| Diabetes mellitus | 1(25%) | 2(50%) | 0.260 |
| TG (mmol/L) | 1.4±0.4 | 1.5±0.5 | 0.648 |
| TC (mmol/L) | 4.2±0.8 | 4.2±0.9 | 0.257 |
| LDL-C (mmol/L) | 2.2±0.6 | 2.4±0.7 | 0.167 |
| HDL-C (mmol/L) | 0.9±0.2 | 1.0±0.3 | 0.352 |
| Mean transvalvular gradient (mmHg) | 15±4.2 | 40±5.0 | <0.001 |
| LVEF (%) | 68±5.4 | 65±5.8 | 0.257 |

Data are expressed as the mean value ± standard deviation or number (%). Comparisons between non-CAVD and CAVD group were analyzed by Student’s t-test or chi-square test. CAVD, calcific aortic valve disease; BMI, body mass index; TG, triglyceride; TC, total cholesterol; LDL-C, low-density lipoprotein cholesterol; HDL-C, high-density lipoprotein cholesterol; LVEF, left ventricular ejection fraction.

**Supplementary figure 1. The identification of human valve interstitial cell.**

**
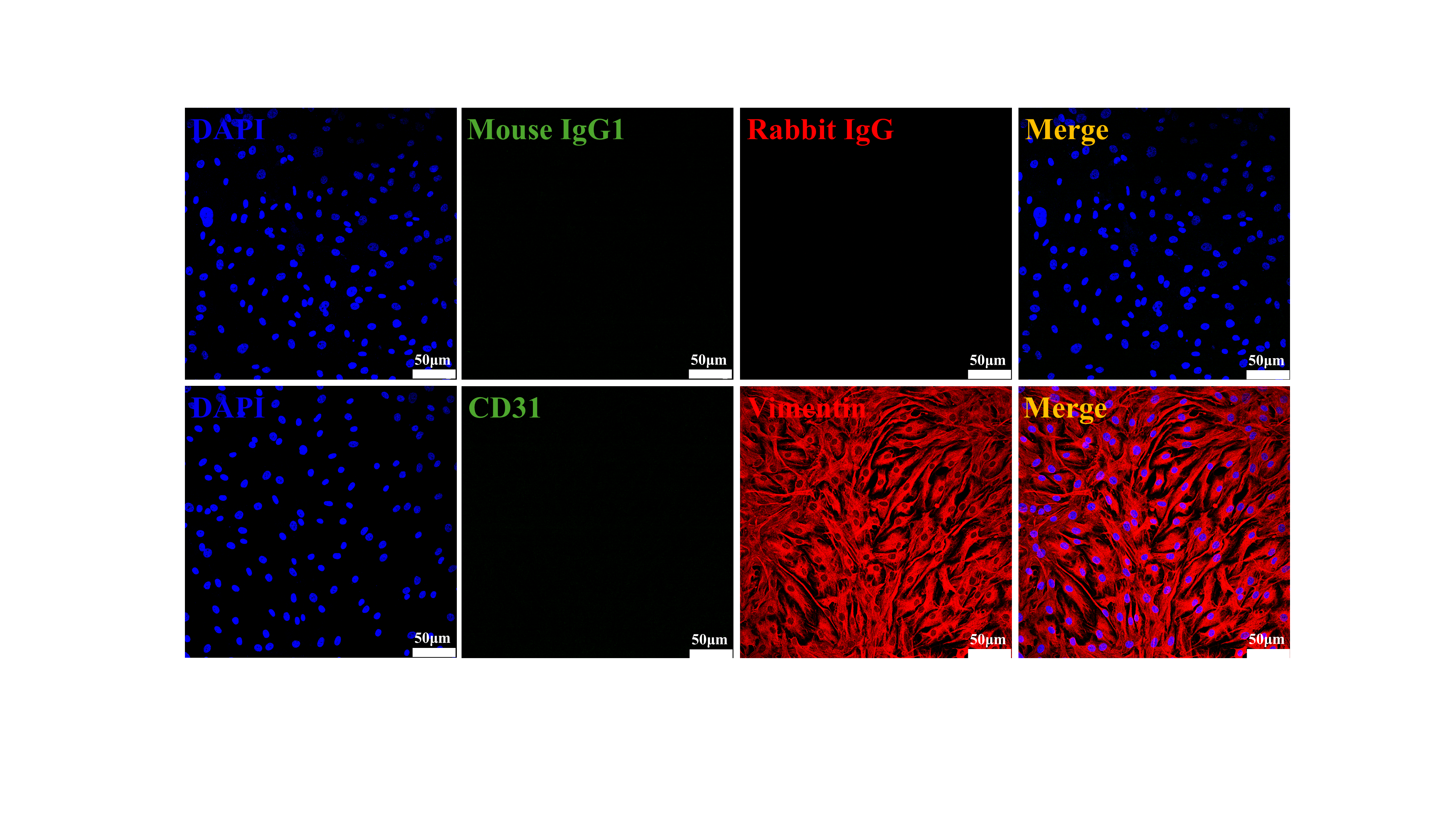
**

**Supplementary figure 2.** **The role of** **TSP-1 silencing on inflammatory cytokines levels *in vitro*.** Data are presented as mean ± SEM and statistical significance were compared by two-way analysis of variance followed by Bonferroni’s multiple comparison test (n=6 for each group). TSP-1, thrombospondin-1; OM, osteogenic induction medium; MCP-1, monocyte chemoattractant protein-1; IL, interleukin; TNF, tumor necrosis factor. NS, not significant; * *P* < 0.05.


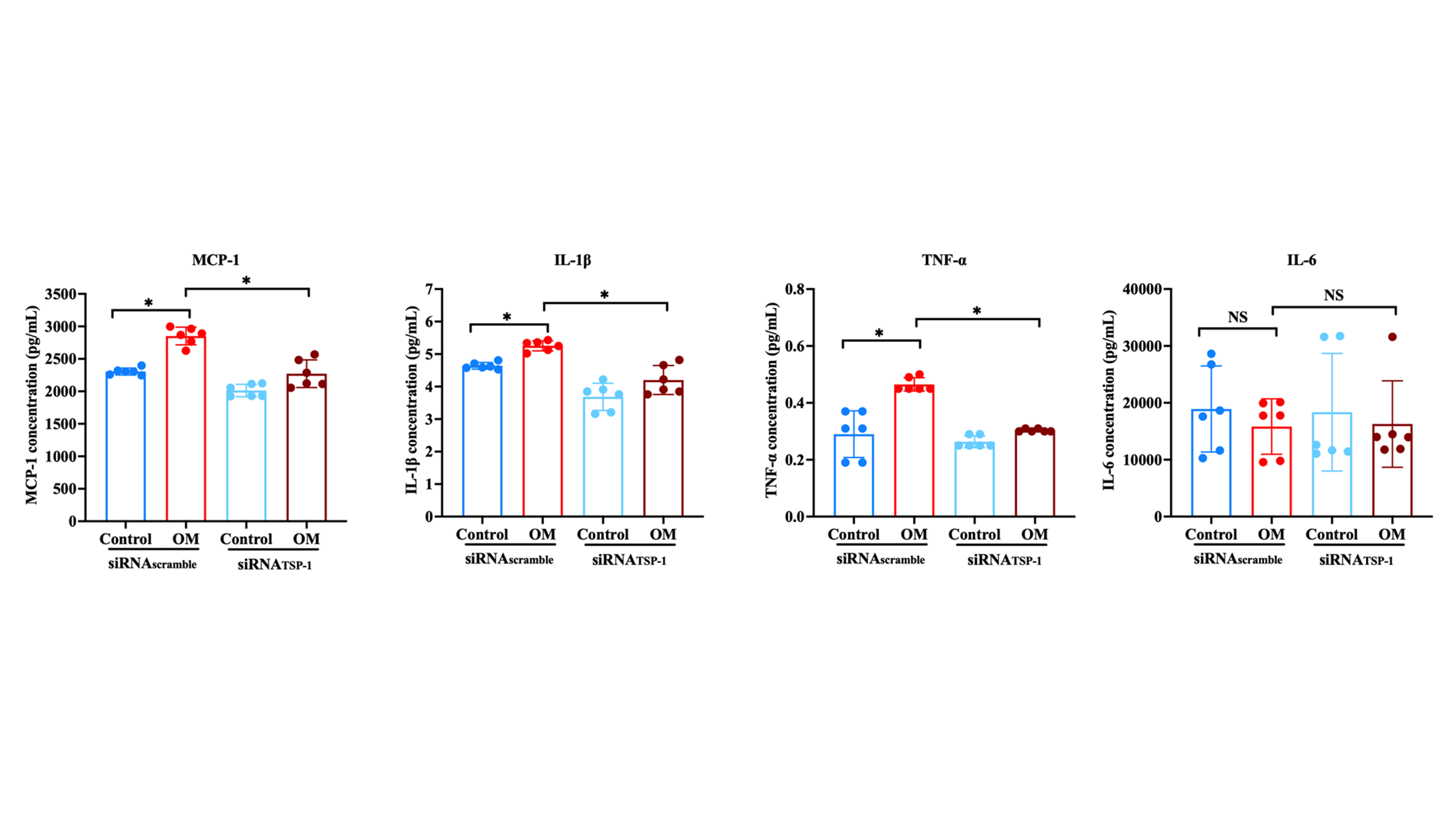


**Supplementary figure 3.** **TSP-1 silencing alleviates osteogenic differentiation of VICs via inhibiting NF-κB pathway.** A. Western blot analysis and quantification of pNF-κB p65 and total NF-κB p65 expression when human VICs were incubated with rhTSP-1 in the presence of osteogenic induction medium with or without PDTC (n=6 for each group). B. Immunofluorescence staining for the nuclear translocation of NF-κB when human VICs were incubated with rhTSP-1 in the presence of osteogenic induction medium with or without PDTC (n=6 for each group). C. Immunofluorescence staining for OPN when human VICs were incubated with rhTSP-1 in the presence of osteogenic induction medium with or without PDTC (n=6 for each group). D. Flow cytometry for OPN when human VICs were incubated with rhTSP-1 in the presence of osteogenic induction medium with or without PDTC (n=4 for each group). Data are presented as means ± SEM and statistical significance were compared by two-way analysis of variance followed by Bonferroni’s multiple comparison test. pNF-κB, phosphorylated nuclear factor-κB; rhTSP-1, recombinant human thrombospondin-1; VICs, valve interstitial cells; PDTC, pyrrolidinedithiocarbamate ammonium; OPN, osteopontin; OM, osteogenic induction medium; DAPI, 4′,6-diamidino-2-phenylindole. NS, not significant; * *P* < 0.05.

**
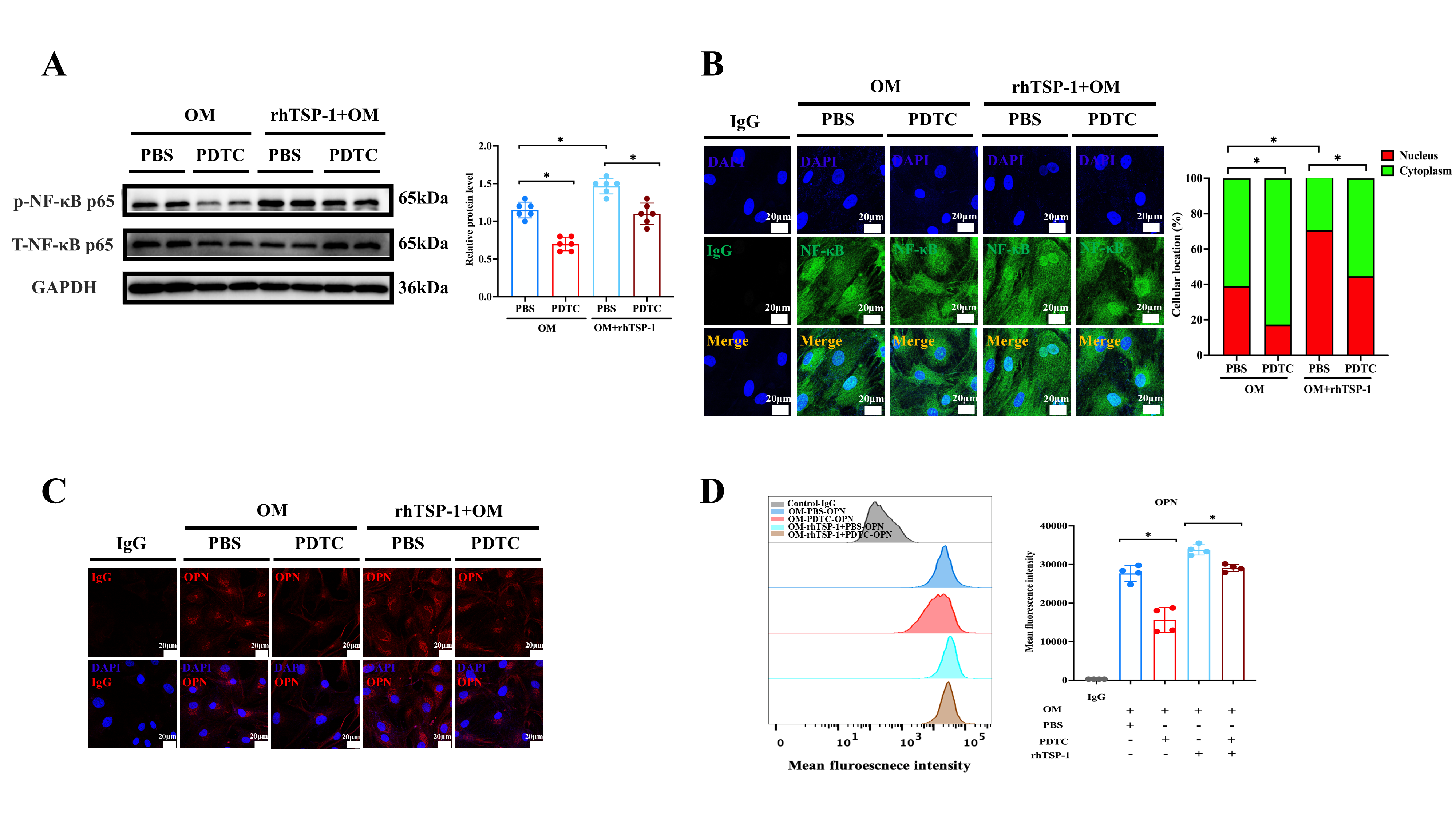
**

**Supplementary figure 4.** **The role of TSP-1 silencing on inflammatory cytokines levels in ApoE^-/-^ mice.** Data are presented as mean ± SEM and statistical significance were compared by two-way analysis of variance followed by Bonferroni’s multiple comparison test (n=5 for ND group, n=8 for HCD group). TSP-1, thrombospondin-1; AAV, adeno-associated virus; ND, normal diet; HCD, high cholesterol diet; MCP-1, monocyte chemoattractant protein-1; IL, interleukin; TNF, tumor necrosis factor. NS, not significant; * *P* < 0.05.

**
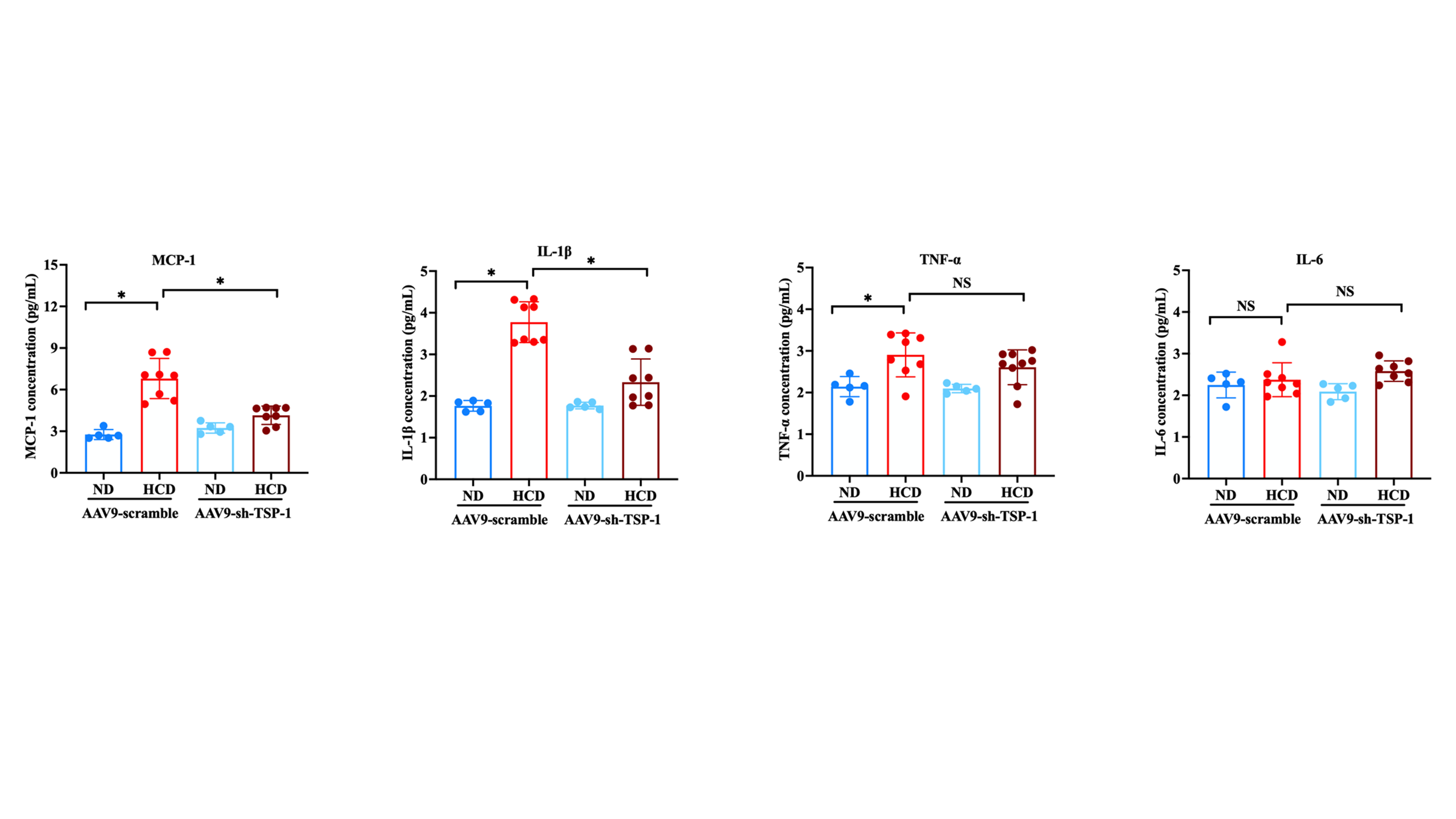
**

**Supplementary figure 5.** **The role of anti-CD47 on aortic valve calcification in ApoE^-/-^ mice.** H&E and Von Kossa staining of aortic valve leaflets and immunohistochemical staining showed TSP-1 in ApoE^-/-^ mice administrated with the inhibitory anti-CD47 antibodies or IgG control. Data are presented as mean ± SEM and statistical significance were compared by student’s t-test (n=5 for each group). CD47, cluster of differentiation 47; IgG, immunoglobulin G; TSP-1, thrombospondin-1; H&E, hematoxylin-and eosin.


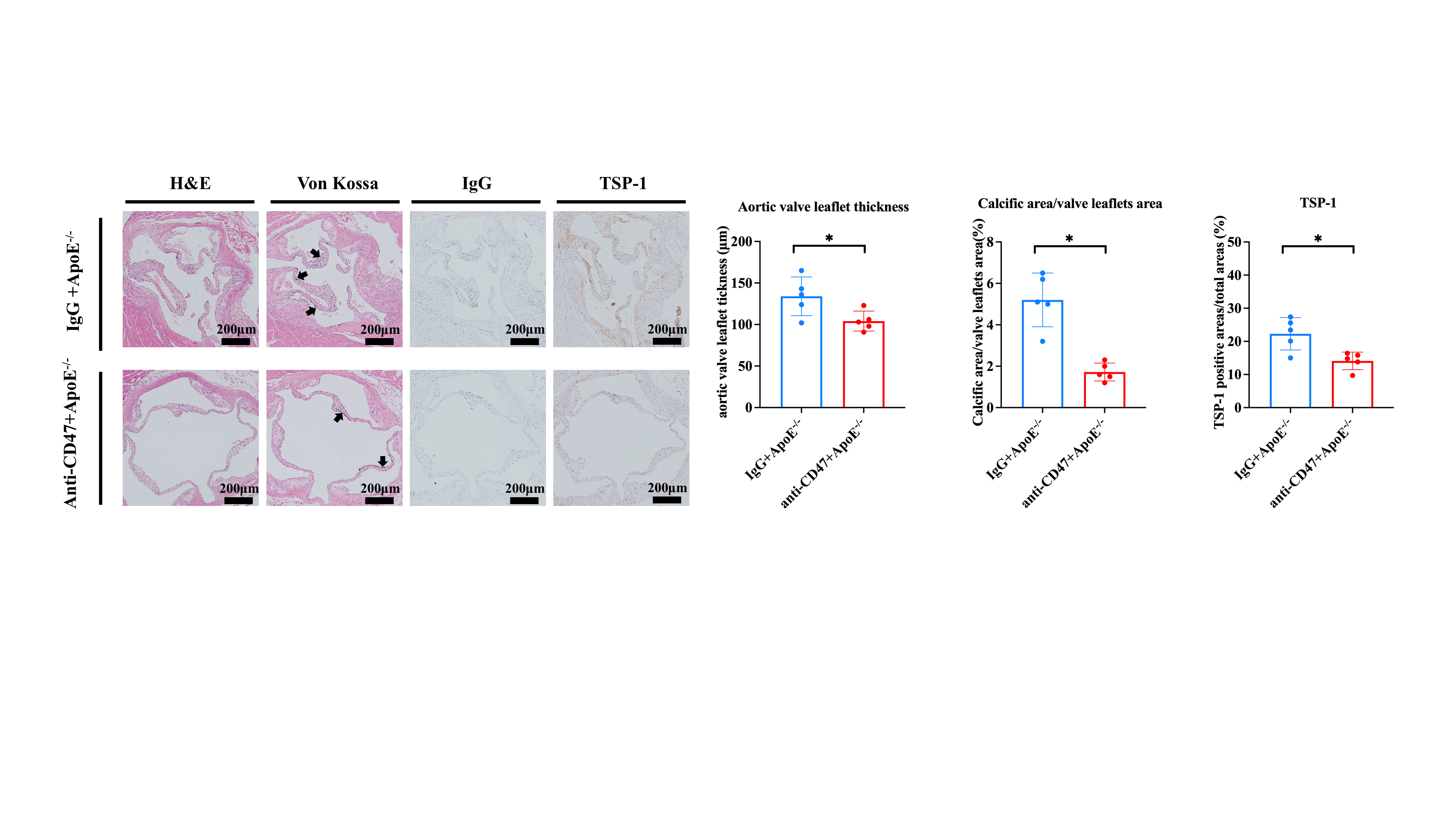

Supplement: Supporting Information — Additional supporting information can be found online in the Supporting Information section. Supporting tables and figures were provided to support our results. Table S1. Clinical characteristics of enrolled participants. Figure S1. The identification of human valve interstitial cell. Figure S2. The role of TSP-1 silencing on inflammatory cytokines levels in vitro. Figure S3. TSP-1 silencing alleviates osteogenic differentiation of VICs via inhibiting the NF-κB pathway. Figure S4. The role of TSP-1 silencing on inflammatory cytokine levels in ApoE−/− mice. Figure S5. The role of anti-CD47 on aortic valve calcification in ApoE−/− mice. [file 3845211.f1.docx]
